# A Randomized Clinical Trial to Evaluate the Effect of Canephron N in Comparison to Ciprofloxacin in the Prevention of Postoperative Lower Urinary Tract Infections after Midurethral Sling Surgery

**DOI:** 10.3390/jcm9113391

**Published:** 2020-10-22

**Authors:** Ewa Rechberger, Tomasz Rechberger, Sara Wawrysiuk, Pawel Miotla, Beata Kulik-Rechberger, Andrzej Kuszka, Andrzej Wróbel

**Affiliations:** 1 2nd Department of Gynecology, Medical Faculty, Medical University of Lublin, ul. Jaczewskiego 8, 20-954 Lublin, Poland; ewarechberger92@gmail.com (E.R.); rechbergt@yahoo.com (T.R.); sara.wawrysiuk@gmail.com (S.W.); wrobelandrzej@yahoo.com (A.W.); 2 Department of Paediatric Propedeutics, Medical Faculty, Medical University of Lublin, ul. A. Gebali 9, 20-091 Lublin, Poland; brechberger@interia.pl; 3 Department of Obstetrics and Gynecology, Medical Faculty, Am Krankenhaus 5, 24211 Preetz, Germany; a.kuszka@klinik-preetz.de

**Keywords:** urinary tract infection, prophylaxis, midurethral sling, ciprofloxacin, phytodrug

## Abstract

Urinary tract infections (UTIs) are one of the most common reasons for antibiotic prescriptions among women worldwide. UTIs are also associated with intra- and postoperative catheterization, which is an essential component of many gynecological surgical procedures, including midurethral sling (MUS) placement. The aim of this study was to compare the incidence of UTI subsequent to a MUS procedure. The study involved 562 female patients who underwent MUS procedures due to stress urinary incontinence (SUI). Patients were assigned in a 1:1 ratio to two study groups: patients receiving 500 mg of ciprofloxacin three times a day for 3 consecutive days after surgery or patients receiving 5 mL of Canephron taken orally three times a day for 3 weeks. After analyzing the collected data, it was found that in the group of patients receiving ciprofloxacin, 29 women (10.98%) had a UTI, whereas in the group of patients receiving Canephron, 36 women (13.64%) had a UTI within 6 months after the patient’s MUS procedure. No statistically significant difference between the two groups was noted. Postoperative prophylaxis with a phytodrug can be perceived as an attractive option in the reduction of antibiotic consumption among female patients after a MUS procedure.

## 1. Introduction

Urinary tract infections (UTIs) are one of the most common reasons for antibiotic prescriptions among women. At least 50% of women will develop one UTI episode during her lifetime. Moreover, it has been observed that 27% of women will develop at least one culture-confirmed recurrence within the 6 months following the initial infection and therapy [1]. The World Health Organization (WHO) currently recognizes antibiotic resistance as one of the biggest threats to global health. According to the data published by the Centers for Disease Control and Prevention, each year in the United States more than 35,000 people die due to antibiotic-resistant infections [2]. UTIs account for about 40% of hospital-acquired infections, and 80% of these infections are associated with urinary catheters compared with only 3–4% per year for women in the general population [3,4]. This pathology is known as a catheter-associated UTI (CAUTI), and it has a daily estimated risk of 3–7% in the acute care setting [5]. Other less commonly reported adverse events associated with bladder catheterization include structural damage to the urethral epithelium, bleeding, creating a false passage, and obvious patient discomfort [6]. However, urinary catheterization is an essential component of many gynecological surgical procedures, including minimally invasive midurethral sling placement, but this obviously increases the risk of nosocomial CAUTI. Although decreasing the duration of catheterization significantly lowers this risk, it can still be as high as 38% in the first 6 weeks following catheter removal, even among women undergoing relatively short-term catheterization for elective gynecological surgery [7,8].

Midurethral slings (MUS) are still considered to be the gold standard in the surgical treatment of stress urinary incontinence (SUI), with an estimated rate of 198.3 per 100,000 person-years annually in the United States [9]. One possible mechanism behind CAUTI after short-term (up to 2 h) catheterization is bacteria entering into the bladder by direct inoculation at the time of catheter insertion; another possibility, though much less likely, is an extraluminal route, by which bacteria ascend from the urethral meatus along the catheter-urethral interface [10,11]. On the other hand, some data indicate that for catheters placed for <3 days, the biofilm growing on the surface of a catheter is not considered to be a reservoir for subsequent UTIs and proper aseptic techniques during catheterization are likely more important in preventing UTIs following short-term catheter placement [12,13].

Nevertheless, bacterial pathogens causing CAUTI during and especially following hospitalization are increasingly resistant to antibiotics and often require complicated treatment with markedly increased costs [14]. Therefore, the introduction into clinical practice of any nonantibiotic regimen is of pivotal importance, as it may decrease the possibility of hospital-acquired UTI during commonly performed surgical procedures and also lead to a decrease in bacterial strains resistant to antibiotics. An effective nonantibiotic approach in UTI prophylaxis remains very attractive for both physicians and patients. One of the potential options to achieve this goal is the usage of Canephron N (Bionorica, Germany), which is a phytotherapeutic drug with diuretic, spasmolytic, anti-inflammatory, antibacterial, and nephroprotective properties [15,16,17]. The main ingredients of the herbal, medicinal product Canephron N are pulverized rosemary leaves, century herbs, and lovage roots. In an in vitro study, the administration of rosemary extracts showed a strong bacteriostatic effect in growth assays for such strains as *Escherichia coli*, *Klebsiella pneumoniae*, *Proteus mirabilis*, *Enterobacter cloacae*, *Citrobacter freundii*, and *Pseudomonas aeruginosa* [18]. Research conducted on animal models showed that after oral administration Canephron is effective in the reduction of inflammation and hyperalgesia in experimental cystitis and hyperalgesia with induced prostatitis. These properties can be the effects of the inhibition of prostaglandin E2 and leukotriene B4 biosynthesis [19]. The effectiveness of Canephron N in the prevention of UTIs in high-risk women undergoing urodynamic examination has been demonstrated [20]. A comparative analysis showed no statistically significant differences in UTI incidence between a group of patients with at least one risk factor for a UTI, who, after the conducted urodynamic study, received a single dose of 3 g of fosfomycin and a group receiving 5 mL of Canephron N three times daily for 1 week. In turn, Wagenlehner et al. assessed the clinical effectiveness of Canephron N in the treatment of UTI by comparing it to the pharmacotherapy, using fosfomycin trometamol. It was shown that only 16.5% of patients receiving Canephron N required additional antibiotic therapy compared with 10.2% of women receiving primary fosfomycin, which proved the non-inferiority of the phytotherapeutic drug to the standard antibiotic therapy [21].

The aim of this study was to compare the effectiveness of the herbal product Canephron N with ciprofloxacin in the prevention of postoperative lower urinary tract infections after the transobturator monofilament sling (T-sling-Hernia Mesh, Italy).

## 2. Materials and Methods

The prospective, time-series study involved 562 patients who underwent T-sling procedures due to stress urinary incontinence (SUI) in a single gynecological center from January 2016 to December 2019. The Institutional Review Board approved the study protocol (KE-0254/74) and all participants gave written informed consent. The diagnosis was based on a clinical examination, which included a detailed interview, including Incontinence Questionnaire-Urinary Incontinence Short Form (ICIQ-SF), Urinary Distress Inventory (UDI-6), and Incontinence Impact Questionnaire Short Form(IIQ-7); a voiding diary; and a gynecological examination with a positive cough test. Simple randomization was used from pseudorandom numbers generated by a computer to allocate patients into the study groups in a ratio of 1:1 (group A—women receiving 500 mg of ciprofloxacin three times daily for 3 consecutive days after surgery; group B—women receiving 5 mL of Canephron N taken orally three times daily for 3 weeks). Researcher E.R. was responsible for proper randomization but was not a member of the operation team. Researchers S.W., P.M., and B.K.-R., who were responsible for assessing the postoperative effectiveness of treatment, were not aware of the assignment of patients to the study groups. Patients were qualified to participate in the study after the exclusion of the presence of other gynecological disorders, such as fibroids, ovarian cysts, or a significant degree of Pelvic Organ Prolapse (only patients with grade 0 or 1 according to Pelvic Organ Prolapse Quantification System (POPQ) were qualified to participate in the study). Additional exclusion criteria from the study included urodynamically confirmed detrusor muscle overactivity and excessive urine retention after voiding (post-void residual—PVR > 100 cm^3^). Moreover, the previous history of recurrent UTI (defined as ≥ 2 per 6 months or ≥ 3 per year) was an additional exclusion criterion. All procedures were performed under short-term general anesthesia (Diprivan) in a standard, previously described way with two additional sutures in order to prevent tape displacement during final tape positioning [22]. Monofilament (Transobturator Tape)TOT-Sling (Herniamesh, Italy) tapes were used for all operations. All study participants received one dose of an intravenous antibiotic cefixime (1 g) 30 min prior to the start of the surgical procedure, including urinary catheter insertion (as per hospital protocol). Cefixime is a new cephalosporin that is more active against enterobacteriaceae than the conventional cephalosporins. About 20% of the drug is excreted by the kidneys as an active drug. For all study patients, we strictly followed the rules of sterile catheterization, which involved the cleansing of the periurethral area for 2 min with an antiseptic solution, wearing sterile gloves, and using a strict no touch, sterile technique, which includes the use of a sterile catheter pack, antiseptic solution, sterile lignocaine, and sterile water to inflate the balloon. The Foley catheter was removed 3 h after the procedure. Patients were asked to spontaneously void when they felt a normal need to urinate after removing the Foley catheter. The residual volume (PVR) was assessed by ultrasound.

Immediately after surgery, all patients received a special leaflet concerning the potential complications of this type of surgery, including all necessary information about behavioral UTI prevention.

The criteria for UTI and bacteriuria used in this study were as follows: for postoperative UTI, a positive dipstick test and a urine culture of ≥10^5^ colony forming units (cfu/mL) or ≥10^4^ cfu/mL with clinical indications of UTI, such as pyrexia (38 °C) or suprapubic tenderness. Participants were advised to contact research staff immediately and to collect a urine specimen should they experience urinary symptoms consistent with a UTI. When participants contacted research staff (E.R.), they were administered a brief structured interview regarding the type and duration of symptoms and referred to their physicians for diagnosis and treatment.

The primary endpoint was the percentage of participants who experienced a clinically diagnosed and treated UTI within 6 months after surgery, whether or not results from a urine culture were available. Diagnosis and treatment were decided by the treating physician. Secondary endpoints included the effectiveness of the treatment by means of a gynecological examination, together with a cough test with a comfortably full bladder (200–250 mL), followed by a sonographic PVR assessment after spontaneous voiding. Moreover, a subjective evaluation of surgery effectiveness was performed by means of ICIQ-SF, UDI-6, and IIQ-7 questionnaires, supplemented by the Patient Global Impression of Improvement (PGI-I), with possible answers including “very much better”, “much better”, “a little better”, “no change”, “a little worse”, “much worse”, and “very much worse”, in order to assess the impact of surgery on pre-existing incontinence [23,24,25].

### Statistical Analysis

The statistically required sample size for effect size w = 0.2 (medium effect size convention) and the 0.95 power of the study was computed using the goodness-of-fit a priori test (G*Power, Düsseldorf, Germany) [26]. The size of the sample was estimated at 495 patients. The obtained results were analyzed statistically with the use of STATISTICA 10.0 PL software [StatSoft Polska Sp. z o.o. Cracow, Poland]. The compliance of the distribution of individual variables within the groups with the normal distribution was checked using the Kolmogorov-Smirnov test with the Lilliefors correction and the Shapiro-Wilk test. Student’s t-test was used to compare two independent groups using interval variables, while the chi^2^ test was used for two independent groups using nominal variables. The one-way ANOVA was used to test the differences between measures. The analysis of the differences between the repeated measurements in pairs was carried out using the Tukey post hoc test. The level of statistical significance of the differences was *p* < 0.05.

## 3. Results

**Figure 1:**
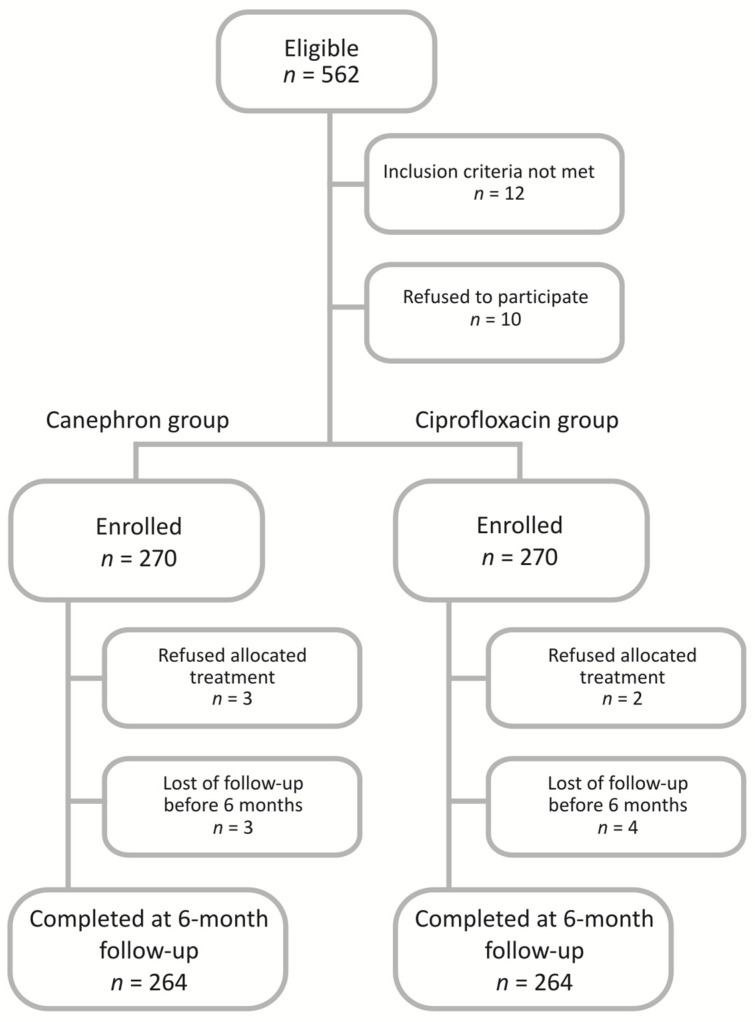
Flowchart of the participants in the study.

The flow of participants through each stage of the study is presented in Figure 1.

Both groups were homogenous across age, type of operation (only TOT outside-in), and severity of illness as indicated by ICIQ-SF questionnaire scores (Table 1).

After analyzing the collected data, it was found that 29 women (10.98%) in the group of patients receiving ciprofloxacin had a UTI, whereas 36 women (13.64%) had a UTI in the group of patients receiving Canephron. Additionally, an interim analysis performed at 3 months after surgery revealed the occurrence of a UTI in 9 (3.4%) patients in the ciprofloxacin group and in 11 (4.1%) women in the Canephron N group. The chi-square test showed that there was no statistically significant difference in the effectiveness of UTI prevention between the two drugs (chi^2^ = 0.86; *p* = 0,35).

The results of the ICIQ-SF, UDI-6, and IIQ-7 questionnaires for both study groups are presented in Table 2 and Table 3.

As expected, the clinical effectiveness of midurethral sling (MUS) procedures was not statistically different across the two investigated groups, as shown by the questionnaire results and also by the Patient Global Impression of Improvement (PGI-I) index. The majority of patients rated the results of their surgery as “very much better” or “much better”, accounting for 84.62% of the ciprofloxacin group and 82.73% of the Canephron N group (chi^2^ test; not significant).

## 4. Discussion

Currently, midurethral slings are the primary choice for SUI surgery performed in women aged 18 to 64 years, whereas all other anti-incontinence procedures are relatively uncommon [9]. Additionally, in Poland, midurethral slings are the most popular surgical SUI treatment [27]. However, given the Food and Drug Administration’s ongoing investigation into the risks of vaginal tapes for SUI surgical treatment, it is critically important for future research to carefully evaluate the short-term risk as well as long-term outcomes of synthetic slings compared to other stress incontinence surgeries. Developing practices to reduce unnecessary UTI risk is a promising area for quality improvement. However, from a public health standpoint, we should be careful not to encourage antibiotic use when it might not be necessary. Therefore, we have tested the hypothesis that herbal medicine (Canephron N) will effectively reduce the risk of a UTI after catheter removal. The meta-analysis of available data indicates that despite the use of antibiotic prophylaxis at the time of removal of a urinary catheter to prevent subsequent urinary tract infections, the estimated rate of such a complication varies from 15% to 18% [28]. This is in agreement with our data, as the prevalence of UTI among patients receiving ciprofloxacin prophylaxis was around 11%. In the randomized phase III non-inferiority clinical trial, the comparison was conducted between Canephron N and fosfomycin trometamol (FT), with corresponding placebos, in the treatment of uncomplicated urinary tract infections. Patients in the FT group (*n* = 334), were treated with 3 g of fosfomycin or a placebo, whereas patients in the Canephron N group (*n* = 325) were given two coated active tablets or a placebo three times daily for 1 week. During the 38 days of the follow-up period, 89.8% participants from the FT group and 83.5% from the Canephron N group did not require any additional antibiotic treatment, confirming the non-inferiority of this herbal product. The most commonly reported treatment-emergent adverse events (TEAEs) were gastrointestinal symptoms reported in 22 patients in the FT group and 13 patients in the Canephron N group, respectively. Only one patient in the Canephron N group developed pyelonephritis of moderate intensity [21]. In another prospective randomized study, the efficacy and safety of Canephron N vs. ciprofloxacin as a monotherapy in the treatment of uncomplicated UTI was studied. After one month of observation, the clinical recovery was 93.75% in the Canephron N group and 91.3% in the ciprofloxacin group. After one month, UTI relapses were noted in 18.8% and 12.5% of patients, respectively [29].

Urinary catheterization, even of a short duration, increases the risk of subsequent UTI. Epidemiological data clearly show that UTIs are responsible for about 40% of nosocomial infections, and the vast majority of these infections are associated with urinary catheters [30]. Overall, 23.6% of hospital patients are catheterized for various reasons [31]. Decreasing catheter duration significantly lowers UTI risk, but the risk is still substantial: as high as 38% in the 6 weeks following catheter removal among women undergoing short-term catheterization for elective gynecological surgery [7,8]. Of course, short-term use of an indwelling urethral catheter is a safe and effective strategy in the maintenance of bladder health during and immediately after MUS surgery and it contributes to improved outcomes indirectly related to bladder integrity. Although cystoscopy is strongly recommended after retropubic sling placement, in clinical practice after a transobturator procedure a cystoscopy is usually performed only in a situation where blood appears in the bladder catheter [32].

In fact, in our study group we did not perform even a single cystoscopy during operation because of the lack of suspicion of bladder injury, such as the clear appearance of urine more than 3 h after surgery. However, the insertion of an indwelling urethral catheter is not without risk of complications since it carries a risk of UTI. It was reported previously that among women undergoing elective urogenital surgery, the 6-week cumulative incidence of a symptomatic UTI following catheter removal is as high as 10–64% [33]. Within hours after insertion, bacteria colonize the urinary catheter surface; the incidence of significant bacteriuria is 5% per day while the catheter remains in place in the urinary bladder. In a prospective, randomized trial a 3-h and a 24-h removal of the urinary catheter and vaginal pack were compared following vaginal prolapse surgery [34]. The authors concluded that there was a tendency for increased infection with longer catheterization and recommended the removal of the catheter and vaginal pack after 3 h with careful postoperative monitoring of the patient. In fact, in our study all patients had the catheter removed within 3 h after the procedure. Foon et al. showed no differences between antibiotic therapy and a placebo in the incidence of symptomatic UTI in the group of patients undergoing short-term catheterization due to urodynamic tests [35]. It has also been proven that Canephron N can be effective in the prevention of UTI in female patients undergoing urodynamic studies (UDSs). Patients who demonstrated mixed urinary incontinence, a neurogenic bladder, or unclear lower urinary tract symptoms (LUTS) were included in the study. Patients with at least one risk factor for UTI development randomly received a single oral dose (3 g) of fosfomycin trometamol or Canephron N (5 mL taken orally three times daily for 7 days). In all randomized patients, urodynamic testing, including cystometry with bladder catheterization, was performed. UTI symptoms were observed in 2.8% of patients receiving the fosfomycin trometamol and in 2.7% of patients in the phytodrug group. There was no difference in any additional adverse events between the investigated groups. Therefore, the authors concluded that the prophylaxis of UTI with Canephron N may be considered a good and safe alternative to antibiotic prophylaxis used after urodynamic testing [20]. Even in the current guidelines regarding prophylaxis it has been noted that non-antibiotic prophylaxis should be prioritized before antibiotic prophylaxis in cases of uncomplicated UTI [36].

Nevertheless, our study has several limitations. The study lacks a placebo group. Additionally, we do not compare the effect of sterile catheter insertion against a clean technique [37]. We also do not compare either latex, silicone, or silver-coated indwelling urethral catheters, although such comparisons can be found in the literature [38,39]. In all of our patients, only commonly used latex catheters were used, and therefore we cannot exclude that the type of catheter might influence the likelihood of urinary bladder infection after MUS surgery. We also do not test any other herbal products that may possibly be effective in decreasing UTI after catheterization (e.g., cranberry products) [40]. However, the design, conduct, and analysis of the trials, as well as the active surveillance for outcomes, were held to the highest standards. Moreover, we strongly believe that the size of the study groups makes it unlikely that the difference in outcomes was due to error (alpha).

## 5. Conclusions

Strategies to decrease the length of time associated with catheterization accompanied by effective preventive measures in order to decrease the probability of CAUTI need to be carefully considered in each case but are promising areas to decrease this morbidity and increase patients’ quality of life. Postoperative prophylaxis with a phytodrug can be perceived as an attractive option in the reduction of antibiotic consumption among female patients qualified for TOT surgeries.

## Figures and Tables

**Table 1:** Patients’ demographic data.

	Canephron Group *n* = 264	Ciprofloxacin Group *n* = 264
Age (years), M ± SD	53.45 ± 11.54	52.76 ± 12.43
BMI (kg/m^2^), M ± SD	28.02 ± 3.95	27.71 ± 4.29
ICIQ Short Form	14.35 ± 4.08	14.98 ± 3.30

BMI—body mass index, Incontinence Questionnaire—Urinary Incontinence Short Form, M—mean, SD—standard deviation.

**Table 2:** The influence of TOT surgery on the results of ICIQ-SF, UDI-6, and IIQ-7 questionnaires in patients receiving Canephron N.

CANEPHRON N group	(T0) Before	(T1) 3 Months	(T2) 6 Months	ANOVA	Post hoc
	Mean ± SD	Mean ± SD	Mean ± SD		
UDI-6	64.03 ± 18.98	14.79 ± 22.21	15.78 ± 22.77	F_(2.526) =_ 641.24 *p* < 0.001	**T1 vs. T2: *p* < 0.001** **T1 vs. T3: *p* < 0.001** T2 vs. T3: *p* = 0.80
IIQ-7	62.82 ± 24.30	14.45 ± 27.11	15.10 ± 28.15	F_(2.526) =_ 470.33 *p* < 0.001	**T1 vs. T2: *p* < 0.001** **T1 vs. T3: *p* < 0.001** T2 vs. T3: *p* = 0.93
ICIQ—Short Form	14.35 ± 4.08	3.06 ± 5.03	3.83 ± 5.77	F_(2.524) =_ 693.34 *p* < 0.001	**T1 vs. T2: *p* < 0.001** **T1 vs. T3: *p* < 0.001** T2 vs. T3: *p* = 0.06

TOT—transobturator tape, ICIQ-SF—Incontinence Questionnaire-Urinary Incontinence Short Form, UDI-6—Urinary Distress Inventory, IIQ-7—Incontinence Impact Questionnaire, Bold font—statistically significance.

**Table 3:** The influence of TOT surgery on the results of ICIQ-SF, UDI-6, and IIQ-7 questionnaires in patients receiving ciprofloxacin.

CIPROFLOXACIN Group	(T0) Before	(T1) 3 Months	(T2) 6 Months	ANOVA	Post hoc
	Mean ± SD	Mean ± SD	Mean ±SD		
UDI-6	67.21 ± 9.68	13.87 ± 0.43	14.60 ± 2.14	F_(2.526)=_ 761.21 *p* < 0.001	**T1 vs. T2: *p* < 0.001** **T1 vs. T3: *p* < 0.001** T2 vs. T3: *p* = 0.89
IIQ-7	64.57 ± 2.26	12.95 ± 6.08	13.76 ± 2.89	F_(2.526)=_ 564.78 *p* < 0.001	**T1 vs. T2: *p* < 0.001** **T1 vs. T3: *p* < 0.001** T2 vs. T3: *p* = 0.89
ICIQ Short Form	14.98 ± 3.30	2.26 ± 5.29	3.00 ± 5.24	F_(2.526)=_ 879.42 *p* < 0.001	**T1 vs. T2: *p* < 0.001** **T1 vs. T3: *p* < 0.001** T2 vs. T3: *p* = 0.07

UDI-6—Urinary Distress Inventory, ICIQ-SF—Incontinence Questionnaire-Urinary Incontinence Short Form, UDI-6—Urinary Distress Inventory, IIQ-7—Incontinence Impact Questionnaire, SD—standard deviation, Bold font—statistically significance.
